# High-Order Multimode Waveguide Interferometer for Optical Biosensing Applications

**DOI:** 10.3390/s21093254

**Published:** 2021-05-08

**Authors:** Yuri Hayashi Isayama, Hugo Enrique Hernández-Figueroa

**Affiliations:** School of Electrical and Computer Engineering (FEEC), University of Campinas (UNICAMP), Campinas 13089-970, SP, Brazil; yuri@decom.fee.unicamp.br

**Keywords:** integrated optics, optical biosensors, label-free detection, optical waveguide devices, multimode interference sensors

## Abstract

A generalization of the concept of multimode interference sensors is presented here for the first time, to the best of our knowledge. The existing bimodal and trimodal sensors correspond to particular cases of those interference sensors. A thorough study of the properties of the multimode waveguide section provided a deeper insight into the behavior of this class of sensors, which allowed us to establish new criteria for designing more sensitive structures. Other challenges of using high-order modes within the sensing area of the device reside in the excitation of these modes and the interpretation of the output signal. To overcome these, we developed a novel structure to excite any desired high-order mode along with the fundamental mode within the sensing section, while maintaining a fine control over the power distribution between them. A new strategy to detect and interpret the output signal is also presented in detail. Finally, we designed a high-order sensor for which numerical simulations showed a theoretical limit of detection of $1.9\times 10^{-7}$ RIU, making this device the most sensitive multimode interference sensor reported so far.

## 1. Introduction

The early detection and diagnosis of diseases is of vital importance, both to manage patients’ conditions and to provide them with adequate treatment. In cancer patients, for instance, early diagnosis has proven to be a very significant factor in increasing the chance of curative treatment and long-term survival [1]. The recent COVID-19 global health crisis also showed the relevance of early detection, since it has been crucial for containing the rapid spread of the disease [2,3]. As the aforementioned pandemic has shown, social distancing and confinement play an important role in flattening the epidemic curve, but thorough tracing and massive population testing should be a priority as well [4].

Achieving early diagnosis in daily medical practice might, nevertheless, be somewhat challenging. Conventional laboratory exams usually present long waiting times and current quick point-of-care testing suffers from low specificity for diseases or disease state [2,3]. Since decision-making is often based on clinical judgement, health professionals are not always equipped with the necessary tools to perform accurate early diagnosis. In light of tthis, to aid professionals acting in the diagnostics area, technological solutions that improve testing speed, sensitivity and specificity are constantly being pursued by the scientific community.

Any biosensor basically consists of three parts: a receptor, a transducer and a detection system. The receptor receives the sample and is responsible for interacting with the analyte, the transducer produces a measurable signal that is related to the concentration of analyte within the sample, and the detection system has the function of converting this measured signal into appropriate information for the user [5,6]. One may classify the type of sensor by considering the working mechanism of the transducer, which allows us to divide them into electrochemical [7,8], thermometric [9], piezoelectric [10], magnetic [11] or optical [12,13,14]. Among these various sensor classes, which present desirable characteristics for supporting the early detection and diagnosis of diseases, the optical sensors correspond to a potential technology that can be used to achieve the goal. They can be designed to be compact, rapid/real-time, very sensitive, and label-free detection devices [4,15,16,17,18]. Optical transducers also present several different types of construction and operating principles, among which we may cite: Surface Plasmon Resonance (SPR) [19,20,21], Localized Surface Plasmon Resonance (LSPR) [22], Raman Spectroscopy [23], Resonators [24], and Interferometers [14,25]. In particular, one type of interferometric optical sensor has received a significant amount attention over the last decade: the multimode interferometric sensor.

The first reported use of mode interference for sensing applications was provided by Gut, et al., in 1999, where they experimented with the pair of fundamental TE-TM modes and the first two TE modes (TE${}_{00}$ and TE${}_{01}$) [26]. In 2011, Zinoviev et al. proposed the bimodal waveguide interferometer (BiMW) using the TE${}_{00}$-TE${}_{01}$ modes as a biosensing device [27]. Since then, several works have been presented, with the aim of developing the original device by means of utilizing a different set of modes in the interferometer (the trimodal waveguide interferometer (TriMW)) [17,28] and by improving the efficiency of light-coupling into the chip itself [16] or the desired waveguide modes [18,25,29]. These works contributed to the development of the sensor, adding incremental improvements to sensitivity and signal-to-noise ratio. However, there has been a lack of deeper studies regarding the fundamental working principle of the sensor, the waveguide aspects which are crucial to determining how the modes will interact with the sample, whether there is a limit to increasing mode order, how to produce sensors qhich are able to effectively employ higher-order modes, etc. In an attempt to address these questions, in this work, we propose, for the first time, a generalization of the concept of the bimodal and trimodal waveguide interferometers through numerical simulations by using higher-order modes within the multimode interferometer. Careful analysis of the multimode waveguide geometry revealed that optimal mode order and the size of the core’s cross-section are intimately related, and can be engineered in order to produce considerably higher bulk sensitivities.

The sections of this paper were organized by each original contribution: first, the generalization of the concept of bimodal and trimodal sensors by using higher-order modes, how it can benefit the device quality, and the bulk sensitivity calculations (comparing with previous works results) are presented, as well as an optimized design for a 4th order sensor with an estimated limit of detection of $1.9\times 10^{-7}$ RIU; second, a novel hybrid method for excitation of the higher-order modes was developed, characterized by efficiently and selectively exciting the TE${}_{00}$ and TE${}_{04}$ modes; third, because the interferometer now has a different electromagnetic field (EM) distribution compared to previous works reported in the literature, a new mechanism for detection is also presented; finally, we draw the conclusions and make the final remarks of the work.

## 2. Multimode Waveguide Interference Sensor

The basic operating principle of the BiMW [26,27,30] and the TriMW [17,18,28] consists of comparing how the fundamental mode and the first- (BiMW) or second-order mode (TriMW) respond to variations in the cladding layer of the sensing waveguide. Initially, since the fundamental mode and the first/second-order mode have different propagation constants (β), a phase shift will be observed between them at the end of the multimode waveguide (MMW). Then, because the EM field distribution of each mode is different, altering the constitution (i.e., the refractive index) of the cladding will result in different variations in each mode’s β. Knowing how the two propagating modes will behave with this change in the cladding, and measuring how much the phase difference between the two modes has been altered, it is, therefore, possible to evaluate the devariation in the refractive index of the cladding with high precision. If the sensing waveguide is subject to biofuncionalization, this interferometer may be employed as a biosensing device [16,17,18,27,28,30]. The motivations for using the second-order propagating mode for the TriMW in [28], in contrast with the first-order mode for the BiMW in [27,30], were: less confinement of the high-order mode and an increase in the region of interaction between the evanescent tail of this mode and the sensing area. Both of these factors contribute to increasing the sensitivity of the device. In this work, we seek to expand this concept by analyzing mode orders higher than the second and assessing how mode confinement and propagation constants change with this increase in the order of the propagating mode.

### 2.1. Higher Order Modes Analysis

Two factors should be considered when trying to improve the BiMW and TriMW sensors: first, the devices operate fundamentally by comparing the propagation constants of two given modes and, hence, it is reasonable to assume that the bigger the $\Delta \beta =\beta_{00}-\beta_{0n}$ (where $\beta_{00}$ and $\beta_{0n}$ are the propagation constants of the fundamental and the high-order mode, respectively) the more sensitive the device should be. Any small relative change in $\beta_{0n}$ will have considerable effects, since Δβ is already naturally large; second, it is desirable that one mode interacts more with variations in the cladding than the other. A parameter that has been constantly and solely used to provide information on how much of this interaction each mode has with the cladding is the confinement factor [27,28]. However, the confinement factor does not provide a full picture of the problem. In this type of sensor, one is interested in the interaction between the EM field with the material in the cladding. As will be presented, in some cases, it is possible to have a situation of very low confinement because a large portion of the mode power is located in the substrate, which does not benefit the sensor in any way. In that way, a better metric would be calculating the percentage of the total power that is concentrated in the cladding. Furthermore, the sensing characteristics of this type of device also depend on the mismatch between the propagation constants of the modes present in the sensing area. Changes in the geometry of the waveguide not only impact the EM field distribution of the modes, but also greatly impact their propagation constants. Thus, in order to investigate the influence of the waveguide’s core geometry in the final sensitivity of the device, these two factors (mode EM field distribution and propagation constant) first have to be considered carefully and simultaneously.

**Figure 1:**
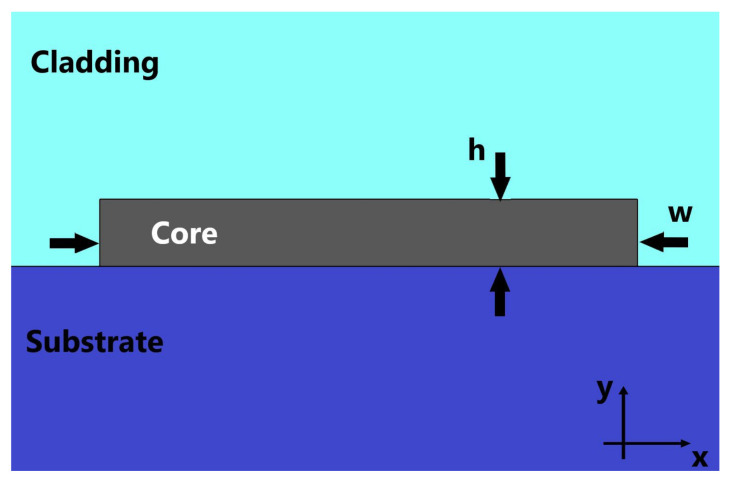
Cross-section view of the multimode waveguide. The waveguide’s core height and width are denoted by h and w, respectively. The propagation is assumed to be in the z-direction.

**Figure 2:**
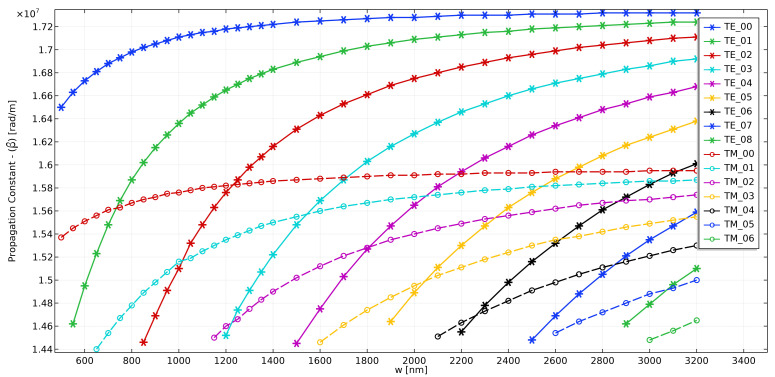
Dispersion relation for TE and TM modes of a Si${}_{3}$N${}_{4}$ channel waveguide with h = 150 nm, SiO${}_{2}$ substrate and n${}_{clad}$ = 1.32.

Our starting point was a Si${}_{3}$N${}_{4}$ (n${}_{{\mathrm{Si}}_{3}N_{4}}$ = 2.0394) channel waveguide, for operation in 633 nm wavelength, with core height (h) of 150 nm, SiO${}_{2}$ substrate (n${}_{SiO_{2}}$ = 1.4570) and a cladding with refractive index n${}_{clad}$ = 1.32 (close to water), as presented in [28]. Figure 1 shows a schematic of the waveguide cross-section. The waveguide’s core width (w) was varied, and the propagation constants of the modes were calculated, as shown in the dispersion relation of Figure 2. The solid lines are the dispersion curves for TE modes and the dashed lines are for TM modes. Due to the aspect ratio of the waveguide (width of the core much larger than its height), TM modes present much smaller propagation constants, as the modes are not well-confined in the core and much of their power propagates through both the cladding and the substrate. All numerical simulations were performed using Comsol Multiphysics^®^.

For TE modes, however, it is interesting to note that, if we compare the fundamental TE mode (TE${}_{00}$ in Figure 2) with other higher-order TE${}_{0n}$ modes when they are close to cutoff condition, as we increase the order of the mode, or the value of *n*, larger values for $\Delta \beta (w=w_{\mathrm{cutoff}})$ are obtained. There is, naturally, a limit to this increase, since the value of $\beta_{00}$ cannot grow indefinitely, which suggests there is an optimal mode order for each waveguide height design. This can be seen in Table 1, showing that, for h = 150 nm, values of Δβ increase until mode TE${}_{07}$. Employing further higher-order modes should not bring any potential improvements in terms of device sensitivity.

The other important aspect to be considered was the distribution of power in the core, cladding and substrate (P${}_{\mathrm{core}}$, P${}_{\mathrm{clad}}$ and P${}_{\mathrm{subs}}$, respectively). The time average of the power flow through a surface *S* is given by

(1) $$P_{S}={\;\int \int \;}_{S}\frac{1}{2}Re\left\{\left(E\times H^{*}\right)\cdot u_{z}\right\}dS,$$

where **E** is the electric field, $H^{*}$ is the complex conjugate of the magnetic field, and $u_{z}$ denotes a unit vector in the z-direction (which is assumed to be the direction of propagation, perpendicular to the surface *S*). The surface *S* can be any of the regions: core, cladding or substrate. Calculating the power flow in each region, one can obtain the power distribution by evaluating

(2) $$P_{\mathrm{region}}\left[\%\right]=\frac{P_{A}}{P_{\mathrm{total}}}\times 100\%,$$

where P${}_{A}$ denotes the power calculated by Equation (1) in the core, cladding or substrate and P${}_{\mathrm{total}}$ denotes the total power. Results are shown in Table 2.

Even though, from Table 1, we observed that Δβ increases until mode TE${}_{07}$ and, thus, an increase in sensitivity is expected, from Table 2, we draw different conclusions. Most of the power of the fundamental TE mode is concentrated in the core of the waveguide, and for higher-order TE modes, less of the power is inside the core. The issue is that a large part of the outside power is concentrated in the substrate, with no use for the sensing device. In fact, the amount of power in the substrate is actually larger than the portion in the cladding.

In [28], Ramirez et al. presented optimized simulations for Si${}_{3}$N${}_{4}$ channel BiMW and TriMW sensors with h = 150 nm. From Table 1 and Table 2, one can see that the chosen value for h indeed suggests a better performance for the first two high-order modes (n = 1, 2) and a poor performance for the rest of them. Therefore, in order to employ modes with an order higher than 2, the height of the waveguide must be adapted.

The same simulations were repeated for a waveguide core height of h = 300 nm, and the results are shown in Table 3 and Table 4. For the new *h* used, the value of Δβ is maximum for the TE${}_{04}$ mode and, once more, decreases if we further increase the order. Analyzing the power distribution of the modes in Table 4, the best values are for modes TE${}_{01}$ and TE${}_{04}$, with 16.4% and 16.2% of the power in the cladding, respectively. Comparing the results with the ones in Table 2, now the power located outside of the waveguide core is mostly concentrated in the cladding region instead of the substrate, even though there is more power within the core itself (for instance, mode TE${}_{04}$ had a confinement factor of 62.1% with h = 150 nm, in comparison to 72.2% with h = 300 nm). It is also worth noting that a significant difference in this scenario is the level of mode confinement seen for the fundamental TE${}_{00}$ mode—in every case, it is over 90%. The direct result of this will be a much weaker interaction between the fundamental mode and the changes in the cladding. In contrast, the power in the cladding for higher-order modes remained stable and, as a consequence, these modes should be much more susceptible to perturbations in the sensing region.

With such an improvement, we may conclude that waveguide height plays an important role in designing higher-order multimode waveguide (MMW) interference sensors, as it is this design parameter that determines which mode order will produce the most sensitive device. Additionally, confinement factor is not the only metric to be pursued when determining the best value for *h*, as the presented results showed. It is also important to determine whether the power outside the core is located in a convenient location.

### 2.2. Bulk Sensitivity Calculations

Once the more fundamental aspects of the MMW sensor were addressed, the next step was to calculate the device’s sensitivity and verify how it compares to other devices of the same type presented in the literature.

The bulk sensitivity is related to the effective index variation of the interfering modes when subject to a bulk change in the refractive index of the sensing region of the interferometer. In other words, variations in the sensing region’s refractive index cause the effective indexes of the interfering modes to change, which, in turn, cause a phase difference between the two of them to arise at the end of the interferometric region. This phase difference (Δϕ) may be calculated by

(3) $$\Delta \phi =\frac{2\pi \Delta n_{\mathrm{eff}}L}{\lambda }$$

(4) $$\Delta n_{\mathrm{neff}}=n_{\mathrm{eff},n}-n_{\mathrm{eff},0},$$

where $n_{\mathrm{eff},n}$ is the effective index of the nth order mode (n = 1 for the BiMW and n = 2 for the TriMW), $n_{\mathrm{eff},0}$ is the effective index of the fundamental mode, *L* is the length of the interferometer sensing area, and λ is the vacuum wavelength at the operating frequency. Then, the bulk sensitivity can be calculated by

(5) $$S_{\mathrm{bulk}}=\frac{\partial (\Delta \phi )}{\partial n_{\mathrm{clad}}}=\frac{2\pi L}{\lambda }\eta_{\mathrm{bulk}}$$

(6) $$\eta_{\mathrm{bulk}}=\frac{\partial (\Delta n_{\mathrm{eff}})}{\partial n_{\mathrm{clad}}},$$

where $n_{\mathrm{clad}}$ is the refractive index of the cladding. The parameter $\eta_{\mathrm{bulk}}$ is called intrinsic bulk sensitivity. Since the MMW interference sensor’s sensitivity is dependent on the device’s length (*L*), $\eta_{\mathrm{bulk}}$ is a good comparative parameter for different sensors within this technology, and will henceforth be used for this purpose.

**Figure 3:**
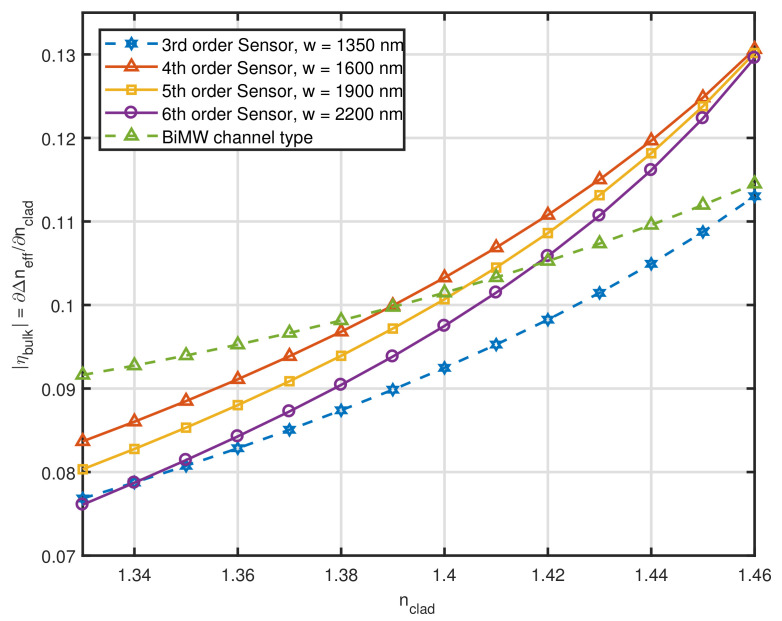
Intrinsic bulk sensitivity as a function of $n_{\mathrm{clad}}$ for Si${}_{3}$N${}_{4}$ channel waveguides with h = 150 nm.

To show that our conclusions in Section 2.1 regarding the Si${}_{3}$N${}_{4}$ channel waveguide with h = 150 nm are consistent, we simulated sensors with mode orders of n = 3, 4, 5 and 6 and compared them to the BiMW channel type of [28]. The results are presented in Figure 3. The BiMW sensor from [28] is represented by the dashed line with open triangles and the other curves correspond to the higher-order sensors simulated. As predicted, the low *h* produced very poor results, worse than even the BiMW for values of n${}_{\mathrm{clad}}$ between 1.33 and 1.39, serving to reinforce that mode delocalization from the core alone does not necessarily translate into highly sensitive devices.

**Figure 4:**
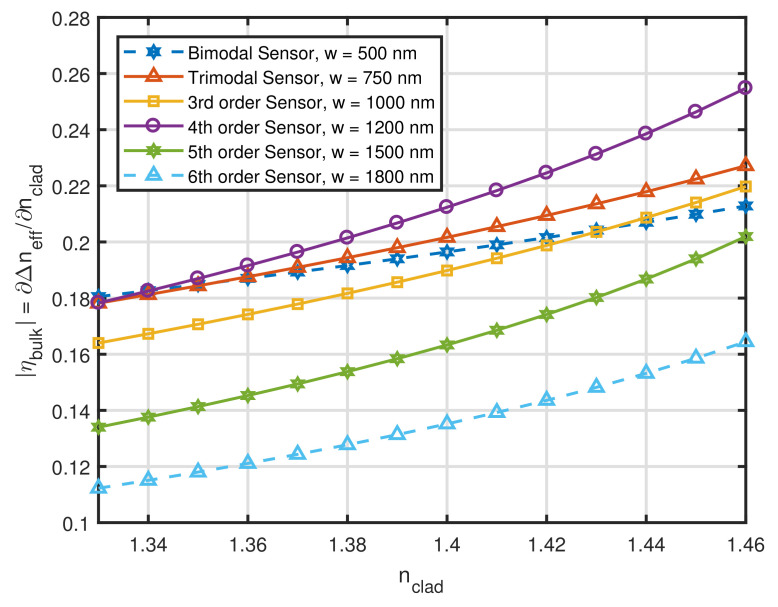
Intrinsic bulk sensitivity as a function of $n_{\mathrm{clad}}$ for Si${}_{3}$N${}_{4}$ channel waveguides with h = 300 nm.

We proceeded to simulate MMW interference sensors with h = 300 nm. Contrary to what happened with *h*, the waveguide width (*w*) should be kept as small as possible, as long as it allows the high-order mode of interest to propagate. The best configurations obtained by numerical simulations are summarized in Figure 4. For these sensors, we note that, once more, the predictions of Section 2.1 were correct. According to Table 3 and Table 4, the mode that presented the highest value for Δβ and, simultaneously, the highest percentage of the EM power in the cladding was the 4th order mode (TE${}_{04}$−$\Delta \beta =4.12\times 10^{6}$ rad/m and P${}_{\mathrm{clad}}=16.2\%$). Figure 4 shows that the 4th order MMW sensor presented the highest intrinsic bulk sensitivity of all designs for practically all values of $n_{\mathrm{clad}}$. In addition, it can be pointed out how $\eta_{\mathrm{bulk}}$ decreased for mode orders superior to 4, which tells us that simply increasing mode order does not necessarily result in a performance improvement for this type of sensor.

**Figure 5:**
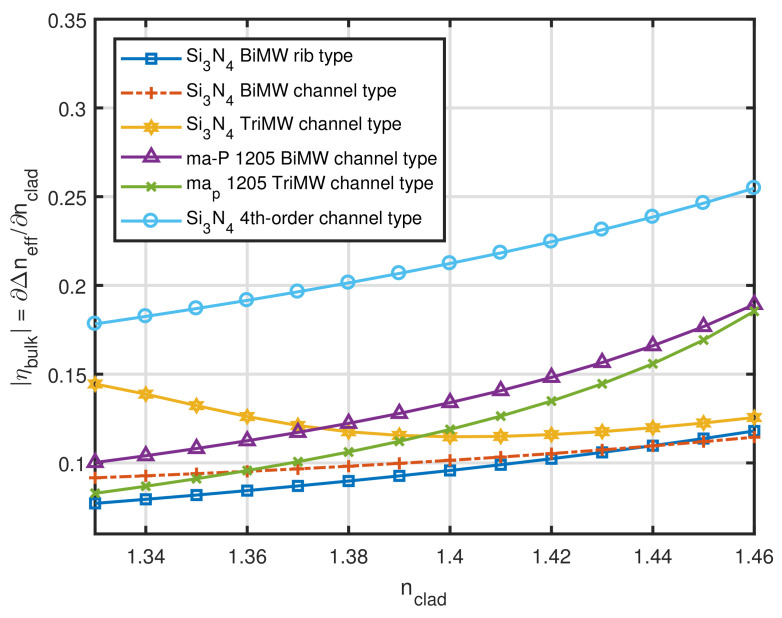
Intrinsic bulk sensitivity as a function of $n_{\mathrm{clad}}$ for the 4th order Si${}_{3}$N${}_{4}$ channel waveguides with h = 300 nm and other sensors in the literature.

Our last step in this analysis was to compare our results with the performances of BiMW and TriMW presented in the literature at this point, as shown in Figure 5. The designs compared in this Figure are the Si${}_{3}$N${}_{4}$ BiMW rib type from [16,27], the Si${}_{3}$N${}_{4}$ and ma-P 1205 BiMW/TriMW of [28], and the Si${}_{3}$N${}_{4}$ 4th order MMW of this work. In terms of intrinsic bulk sensitivity, the Si${}_{3}$N${}_{4}$ 4th order MMW is far superior to all of its peers, being 23.4% more sensitive than the Si${}_{3}$N${}_{4}$ TriMW channel type @n${}_{\mathrm{clad}}$ = 1.33, 58.5% more sensitive than the ma-P 1205 TriMW channel type @n${}_{\mathrm{clad}}$ = 1.40 and 34.6% more sensitive than the ma-P 1205 TriMW channel type @n${}_{\mathrm{clad}}$ = 1.46. Thus, for the whole simulation window of $1.33\leq n_{\mathrm{clad}}\leq 1.46$, the proposed sensor presented a much higher bulk sensitivity.

Other works also managed to improve the BiMW and TriMW sensitivities through the incorporation of better excitation structures [16,18,29,30]. Since the device bulk sensitivity, according to Equation (5), depends on the length (*L*) of the sensing area, sensors with different lengths will produce different sensitivities as well, as stated before. The existing MMW interference sensors in the literature and their bulk sensitivity per sensor length (i.e., $S_{bulk}/L$) are summarized in Table 5. Once more, the 4th order sensor proposed in this work performs at a higher level than the other devices reported to date.

A good metric to compare the presented sensor with other devices from other technologies is the limit of detection (LOD), which tells us the smallest variation in the refractive index of the cladding that could be measured, and can be calculated by

(7) $$\mathrm{LOD}=\frac{3\cdot N/S}{S_{\mathrm{bulk}}},$$

where N/S is the noise-to-signal ratio. To date, the BiMW of [27] presents the best value for LOD in the literature. Even though other devices did present higher bulk sensitivities per sensor length (as seen in Table 5), because of the limited device length or worse N/S ratios, their LOD was not as good as the one reported in [27]. As the experimental apparatus for testing our 4th order sensor is basically the same as the one described in [27], in terms of light source, in-chip light coupling and detection instruments requirements, it is reasonable to assume the same 3N/S $=5\times 10^{-4}$2π rad and a sensor length of L = 15 mm. Doing this, for samples with a refractive index around 1.33, the estimated LOD for the 4th order Si${}_{3}$N${}_{4}$ MMW sensor is $1.9\times 10^{-7}$ RIU (compared to $2.5\times 10^{-7}$ RIU reported in [27]), while, for refractive indexes close to 1.46, the LOD becomes $1.3\times 10^{-7}$ RIU.

## 3. Multimode Waveguide Excitation

After establishing that our 4th order MMW sensor has a good sensitivity level, the next step was to determine how the desired modes can be excited. There are two requirements for this matter: exciting the fundamental and 4th order TE modes, and guaranteeing that these are the sole propagating modes within the multimode waveguide (sensing region) of the sensor.

The first requirement is not very difficult to neet on its own, as it would be possible to use the same strategy employed for BiMW and TriMW sensors, which corresponds to an abrupt transition from a single-mode section to a multimode section. Depending on the desired mode parity, the lateral offset between the two sections may be designed to preferentially excite a certain propagation mode [27,28,30]. However, when moving towards higher-order modes, this excitation strategy becomes inefficient, because the input power coming from the single mode waveguide will be split between several propagating modes. In the BiMW from [16,27,30], the abrupt vertical transition is capable of exciting the fundamental and all modes with odd symmetry, but the multimode waveguide only supports the first odd mode. In the TriMW from [17,18,28], the transition was designed to excite the fundamental and all the even symmetry modes, but, again, the only even modes supported by the multimode section are the fundamental and the second-order TE modes. Should we increase the dimensions of the multimode waveguide to support higher-order modes, this excitation strategy would divide the input power into several undesired modes. Some proposals were presented to increase the efficiency of light coupling into the first- and second-order modes [18,29], but higher order modes were not contemplated.

Since it is not possible to precisely control how the input power will be divided in the multimode section solely by means of an abrupt transition, the second requirement for the MMW sensor is slightly more challenging to accomplish. It is desirable to have only two propagating modes within the sensing region because the interferometric signal at the output of the MMW sensor will be much simpler to translate into a variation in the refractive index. Using several modes will make the resulting interferometric response much more complex and, if we add the future instrumentation noise, the gains of using higher-order modes could easily be lost.

Ebihara et al. [18] proposed a dual single-mode waveguide (DSMW) scheme to excite the TriMW sensor, composed of two single-mode waveguides, with appropriately designed width and an abrupt transition for enhancing coupling efficiency. They reported a high degree of control of the power distribution between the fundamental and second-order TE modes. One advantage was that, by making the power distribution in the fundamental and second-order TE modes very close to 50%:50%, the interferometric signal generated by the TriMW presented a much higher extinction ratio, which is important for suppressing the influence of noise [18,29]. Therefore, it is desirable to have only two propagating modes in the multimode section, as well as keeping their power levels as close as possible.

Here, we propose a similar approach to [18], but with the difference of the incorporation of an additional pair of waveguides to make the excitation of desired modes in the MMW sensor both efficient and controlled. It is not possible to solely excite the fundamental and high-order (4th) modes using DSMW as in [18]. However, our proposed scheme completely suppresses the intermediate modes, increasing the extinction ratio of the output signal and, thus, not only makes feasible higher-order MMW sensors but further decreases the device’s susceptibility to noise.

**Figure 6:**
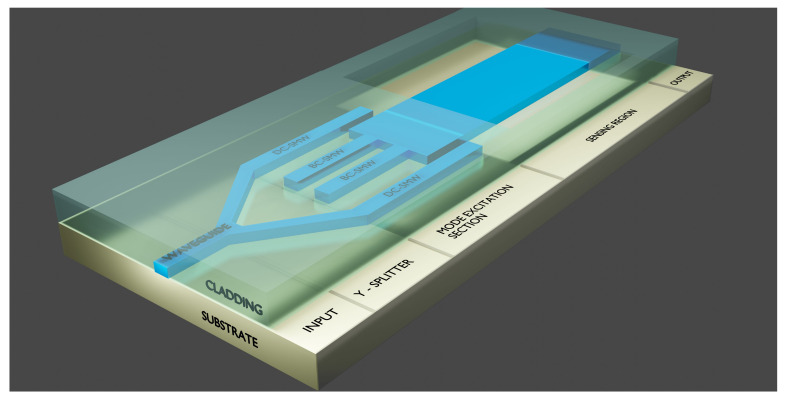
Schematic illustration of a Multimode Waveguide (MMW) Interference sensor.

A schematic of the proposed sensor can be seen in Figure 6. Light is coupled into a single-mode waveguide in the input section, which passes through a Y-splitter, dividing the input power equally between two new singl- mode waveguides, which we shall call directional coupling single-mode waveguides (DC-SMWs) henceforth. Another pair of single-mode waveguides are constructed between the DC-SMWs, which will be called butt-coupling single-mode waveguides (BC-SMWs). Part of the light from the DC-SMWs is transferred to these BC-SMWs and will be inserted in the multimode waveguide via butt-coupling mechanism. The rest of the input power, within the DC-SMWs, will be inserted in the MMW via directional coupling mechanism. By designing the DC-SMW width, it is possible to solely excite the desired high-order mode and by choosing properly the separation between the BC-SMWs, it is also possible to excite only the fundamental mode of the MMW. As will be shown, the power coupled to the MMW is equally divided between the fundamental and the high-order modes without exciting other modes. In the sensing region, the upper cladding that covers the device is removed, and this is where the sample to be tested will be introduced and put in contact with the sensor. Lastly, light will follow to the output section, where it will be captured by detection devices and interpreted.

**Figure 7:**
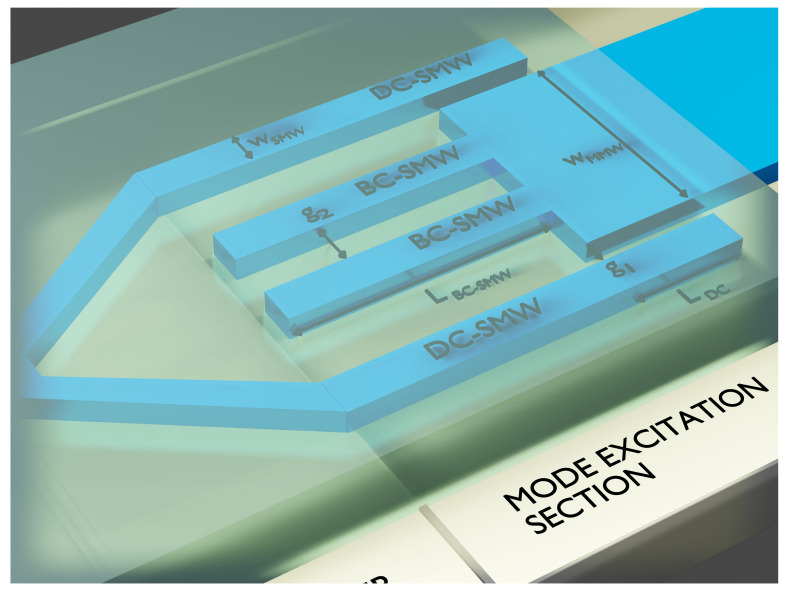
BC-SMW/DC-SMW excitation scheme. w${}_{\mathrm{SMW}}$ is the width of both BC-SMWs and DC-SMWs, w${}_{\mathrm{MMW}}$ is the width of the MMW sensor, g${}_{1}$ is the gap between the DC-SMW and the MMW, g${}_{2}$ is the separation between the BC-SMWs, L${}_{\mathrm{BC}-\mathrm{SMW}}$ is the length of the BC-SMWs, and L${}_{\mathrm{DC}}$ is the length where the DC-SMWs couple to the MMW (the total length of the DC-SMW is L${}_{\mathrm{BC}-\mathrm{SMW}}+L_{\mathrm{DC}}$).

In Figure 7, there is a scheme of the excitation structure, where w${}_{\mathrm{SMW}}$ is the width of both BC-SMWs and DC-SMWs, w${}_{\mathrm{MMW}}$ is the width of the MMW sensor, g${}_{1}$ is the gap between the DC-SMW and the MMW, g${}_{2}$ is the separation between the BC-SMWs, L${}_{\mathrm{BC}-\mathrm{SMW}}$ is the length of the BC-SMWs, and L${}_{\mathrm{DC}}$ is the length where the DC-SMWs couple to the MMW (the total length of the DC-SMW is L${}_{\mathrm{BC}-\mathrm{SMW}}+L_{\mathrm{DC}}$). We divided the analysis for each type of coupling employed and presented the numerical results for the complete structure afterwards.

### 3.1. Directional Coupling Single Mode Waveguides (DC-SMWs)

In order to excite the high-order mode, the 4th order mode for the proposed MMW sensor, directional couplers were utilized. This type of excitation allows for good control over which mode is coupled to the MMW. There are two basic parameters to take into account: the propagation constant of the DC-SMW and the gap between the single- and the multimode sections. From Coupled Mode Theory (CMT) [31], it is known that the coupling of two modes depends on their phase-matching (Δβ = $\beta_{2}-\beta_{1}$, where $\beta_{1}$ and $\beta_{2}$ are the propagation constants of the two modes in question) and the degree of interaction between their EM fields.

If the phase-matching condition is fulfilled (i.e., Δβ = 0), the coupling between the two modes is maximized. Modes that do not satisfy phase matching conditions become uncoupled and exchange little to no power. Considering this fact, the DC-SMW width has to be chosen so the propagation constant of the waveguide exactly matches the propagation constant of the 4th order mode of the MMW. This will result in a strong coupling and the power from the DC-SMWs will be transferred completely to the 4th order mode, not exciting any other mode in the process. We decided to maintain the same waveguide height as the MMW, to simplify future fabrication processes.

Furthermore, the interaction between the EM fields of the two modes in question is defined by the separation between the DC-SMW and the MMW. As they become closer to each other, their evanescent tails start to interact and the mode coupling grows stronger. By choosing the appropriate separation gap, power will be more efficiently transferred from the DC-SMW to the MMW, to the point where all the optical power can be coupled to the MMW within a short length.

### 3.2. Butt-Coupling Single Mode Waveguides (BC-SMWs)

Since the 4th order mode will be excited by means of a pair of directional couplers, the objective of the two BC-SMWs is to excite only the fundamental mode of the MMW sensor. As the BC-SMWs will receive power from the DC-SMWs (also through directional coupling), we chose to keep the single mode waveguide widths the same. The separation between a pair DC-SMW/BC-SMW determines the necessary length of the excitation section.

Again, from CMT, how efficiently a given mode will be excited through a butt-coupling mechanism is determined by the overlap integral between the modes of the BC-SMW and the MMW [31]. Therefore, in order to maximize the power coupled to the fundamental TE mode of the MMW, one must design the BC-SMWs to present a similar EM field distribution. As design constraints, the width of the BC-SMWs is fixed and the waveguides are built symmetrically with respect to the MMW. Therefore, the only design parameter is the separation (gap) between the waveguides.

If both excitation schemes present similar efficiency, the input power can be divided equally between all four single-mode waveguides, and the expected power distribution between the two modes in the MMW will be 50%:50%.

### 3.3. Numerical Results

**Figure 8:**
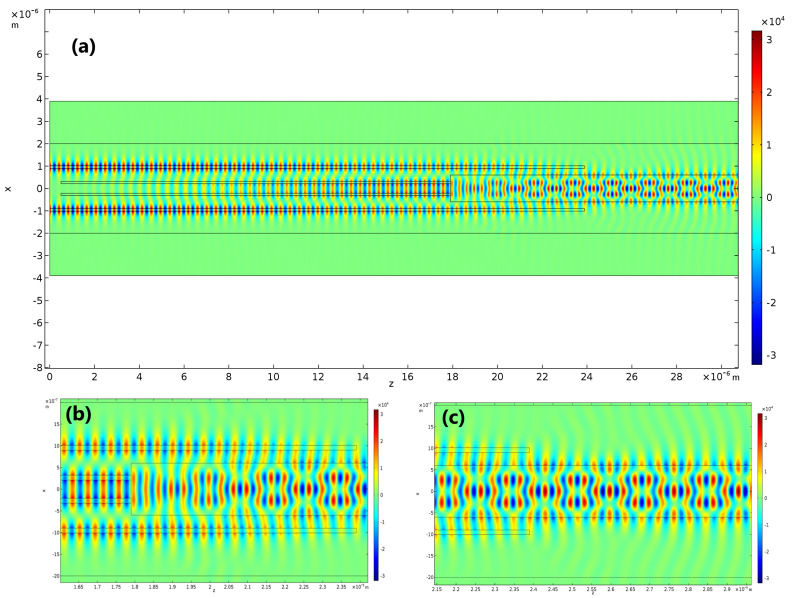
Electric field distribution (E${}_{x}$ component) for the simulated 4th order Si${}_{3}$N${}_{4}$ MMW. (**a**) Simulation starting from the two single-mode waveguides originated from the Y−splitter of Figure 6 up to the sensing area. (**b**) Detail of the excitation section, where the BC−SMWs and DC−SMWs transfer power to the TE${}_{00}$ and TE${}_{04}$ modes. (**c**) Detail of the propagating field in the sensing area of the device.

Taking the information from the previous sections into consideration, we optimized the single-mode waveguides’ width, lengths and separation gaps using a 2D Finite Element Method (2D-FEM) with Comsol Multiphysics^®^. To take the finite height of the waveguides into account, an effective index method modelling was also employed [31]. The results of parameter optimization are shown in Table 6 and, in Figure 8, the electric field distribution is presented. The total length of the excitation section obtained is L${}_{\mathrm{BC}-\mathrm{SMW}}+L_{\mathrm{DC}}$ = 23.9 µm, which is very short compared to the typical complete length of this type of sensor (in the order of 2 cm), and does not burden the device’s size.

In Figure 8a, the simulation started after the Y-splitter seen in Figure 6, where the input power is divided equally between two single-mode waveguides (DC-SMWs). As their core width is small, they present a considerable mode de-localization, allowing the input power to be coupled to the two BC-SMWs, even though the separation gap between them is considerable. In Figure 8b, the excitation of the modes in the multimode section of the sensor is shown in detail. At this point, roughly half of the input power is in the DC-SMWs and the other half is in the BC-SMWs. Again, because the core width of the BC-SMWs is small, the mode de-localization of these two waveguides produce a spread electric field distribution, similar to the field distribution of the fundamental mode of the MMW and, thus, have a good coupling efficiency to this particular mode. Figure 8c presents the field distribution in the sensing area, where one can see the interferometric pattern produced. Overlap integrals were evaluated within the sensing area to measure how much power was coupled to each of the modes in the MMW, and the resulting power distribution obtained is represented in Table 7.

The results in Table 7 show that the devised excitation mechanism is highly effective and efficient, as it not only delivers precisely balanced power between the two desired modes (suppressing all the rest), but also presents a low power loss during the excitation process (4.16%). Guaranteeing that both modes have the exact same power is important when aiming to increase the extinction ratio and, consequentially, make the sensor more robust in terms of noise. If one is interested in using even higher order modes, this excitation method represents a good technical solution, as it is also size efficient.

## 4. Detection Method

A difference between the 4th order Si${}_{3}$N${}_{4}$ MMW and the previous BiMW and TriMW devices is the signal detection. Since the BiMW operates with the first two propagating modes, the detection can be performed with two photodiodes and their signal can be post-processed [16,27,30]. The TriMW resolves the post-processing needed by the BiMW within the physical layer by utilizing an abrupt transition at the end of the sensing region and recovering the interferometric signal at the output single-mode waveguide [17,28]. We decided to use a similar approach to the BiMW to handle the detection. As the mode order increases, it is no longer possible to achieve a good output signal quality with only two photodetectors, because the higher-order modes themselves have more than two intensity peaks. By using only two photodetectors, important information regarding the phase difference between the propagating modes would be lost, and this would have a direct impact on the experimental sensitivity.

For simplicity, assuming that the waveguide is infinite in the vertical direction (*y*-direction in Figure 1), inside the MMW, i.e., $\left|x\right|\leq \frac{W}{2}$, the electric field of the m${}^{\mathrm{th}}$ mode is given by [31]

(8) $$\left\{\begin{array}{c} E_{x}^{m}\left(x,z\right)=A_{m}cos\left(\frac{2u_{m}}{W}x-\frac{m\pi }{2}\right)e^{-j\beta_{m}}\\ u_{m}\approx \left(m+1\right)\frac{\pi }{2}, \end{array}\right.$$

where $A_{m}$ is the amplitude of the m${}^{\mathrm{th}}$ order mode and $\beta_{m}$ is its propagation constant. The TE${}_{04}$ mode has maximum field intensity ($|E_{x}|$) at *x* = $\left\{-\frac{2}{5}W,-\frac{W}{5},0,\frac{W}{5},\frac{2}{5}W\right\}$. At these points and for $z=L_{\mathrm{MMW}}$ (at the end of the MMW, or the output), the total electric field is given by

(9) $$\left\{\begin{array}{c} E_{x}\left(-\frac{2}{5}W,L_{\mathrm{MMW}}\right)=-A_{0}cos\left(\frac{2\pi }{5}\right)e^{-j\phi_{0}}-A_{4}e^{-j\phi_{4}}\\ E_{x}\left(-\frac{W}{5},L_{\mathrm{MMW}}\right)=-A_{0}cos\left(\frac{\pi }{5}\right)e^{-j\phi_{0}}-A_{4}e^{-j\phi_{4}}\\ E_{x}\left(0,L_{\mathrm{MMW}}\right)=A_{0}e^{-j\phi_{0}}+A_{4}e^{-j\phi_{4}}\\ E_{x}\left(\frac{W}{5},L_{\mathrm{MMW}}\right)=A_{0}cos\left(\frac{\pi }{5}\right)e^{-j\phi_{0}}+A_{4}e^{-j\phi_{4}}\\ E_{x}\left(\frac{2}{5}W,L_{\mathrm{MMW}}\right)=A_{0}cos\left(\frac{2\pi }{5}\right)e^{-j\phi_{0}}+A_{4}e^{-j\phi_{4}}, \end{array}\right.$$

where $\phi_{m}=\beta_{m}L_{\mathrm{MMW}}$. The power of each of those peaks is proportional to $|E_{x}{|}^{2}$

(10) $$\left\{\begin{array}{c} P_{2}^{\left(-\right)}={\left|E_{x}\left(-\frac{2}{5}W,L_{\mathrm{MMW}}\right)\right|}^{2}=A_{0}^{2}cos^{2}\left(\frac{2\pi }{5}\right)+A_{4}^{2}-2A_{0}A_{4}cos\left(\frac{2\pi }{5}\right)cos\left(\Delta \phi \right)\\ P_{1}^{\left(-\right)}={\left|E_{x}\left(-\frac{W}{5},L_{\mathrm{MMW}}\right)\right|}^{2}=A_{0}^{2}cos^{2}\left(\frac{\pi }{5}\right)+A_{4}^{2}+2A_{0}A_{4}cos\left(\frac{\pi }{5}\right)cos\left(\Delta \phi \right)\\ P_{0}={\left|E_{x}\left(0,L_{\mathrm{MMW}}\right)\right|}^{2}=A_{0}^{2}+A_{4}^{2}-2A_{0}A_{4}cos\left(\Delta \phi \right)\\ P_{1}^{\left(+\right)}={\left|E_{x}\left(\frac{W}{5},L_{\mathrm{MMW}}\right)\right|}^{2}=A_{0}^{2}cos^{2}\left(\frac{\pi }{5}\right)+A_{4}^{2}+2A_{0}A_{4}cos\left(\frac{\pi }{5}\right)cos\left(\Delta \phi \right)\\ P_{2}^{\left(+\right)}={\left|E_{x}\left(\frac{2}{5}W,L_{\mathrm{MMW}}\right)\right|}^{2}=A_{0}^{2}cos^{2}\left(\frac{2\pi }{5}\right)+A_{4}^{2}-2A_{0}A_{4}cos\left(\frac{2\pi }{5}\right)cos\left(\Delta \phi \right), \end{array}\right.$$

with $\Delta \phi =\phi_{0}-\phi_{4}$ representing the phase difference between the modes TE${}_{00}$ and TE${}_{04}$ after a propagation length of L${}_{\mathrm{MMW}}$. Whenever there is a variation in the refractive index of the cladding, caused by a change in the substance present in the sensing area of the device, there will be a variation in the propagation constants of the two modes. The direct result will be different values for the phases of the modes TE${}_{00}$ and TE${}_{04}$ after the propagation length L${}_{\mathrm{MMW}}$ ($\phi_{0}$ and $\phi_{4}$, respectively) which translate into a change in the phase difference Δϕ. Since the power P${}_{0}$ of Equation (10) is proportional to cos(Δϕ), by measuring the power of the central peak, it is possible to relate the variation in the refractive index of the cladding to the variation in the measured power, equivalent to methodology presented in [30]. However, measuring only the power of the central peak brings a problem, which is dependent on the input power. Therefore, if, for instance, the light source suffers power fluctuations, P${}_{0}$ will be directly affected, leading to incorrect interpretations of the refractive index changes. To solve this problem, we may manipulate the five powers in Equation (10), defining a power signal, *I*, as follows

(11) $$\begin{array}{cc} I & =\frac{\left(P_{1}^{\left(+\right)}+P_{1}^{\left(-\right)}\right)-\left(P_{2}^{\left(+\right)}+P_{2}^{\left(-\right)}\right)}{P_{1}^{\left(+\right)}+P_{1}^{\left(-\right)}+P_{0}+P_{2}^{\left(+\right)}+P_{2}^{\left(-\right)}}=\\ \; & =\frac{A_{0}^{2}\left(cos^{2}\frac{\pi }{5}-cos^{2}\frac{2\pi }{5}\right)+2A_{0}A_{4}\left(cos\frac{\pi }{5}+cos\frac{2\pi }{5}\right)cos\Delta \phi }{\frac{5}{4}A_{0}^{2}+\frac{3}{2}A_{4}^{2}}. \end{array}$$

The constructed signal *I* is of the form $I=C_{1}+C_{2}cos\Delta \phi$ ($C_{1}$ and $C_{2}$ are constants), which is still proportional to the cosine of the phase difference between the two interfering modes of the sensor and, thus, can still be used to establish the relationship with refractive index changes in the cladding in the same way as before. The advantage is that, in Equation (11), the denominator $P_{1}^{\left(+\right)}+P_{1}^{\left(-\right)}+P_{0}+P_{2}^{\left(+\right)}+P_{2}^{\left(-\right)}$ represents the total power in the peaks, serving as a normalization of the output signal. In this way, because *I* is normalized, variations in the input power affect the quality of the measurements much less, eliminating the issues of using simply *P*${}_{0}$.

**Figure 9:**
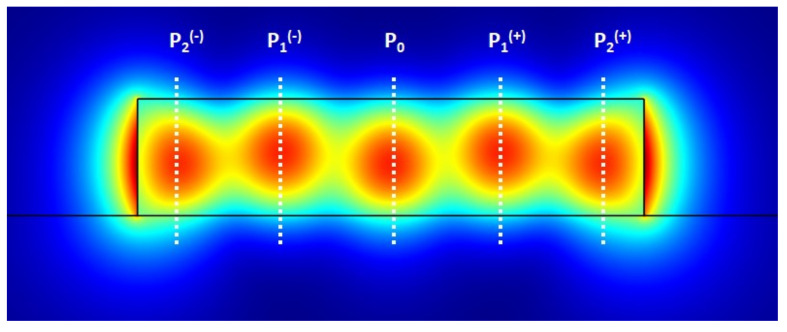
Example of the output signal at the end of the 4th order Si${}_{3}$N${}_{4}$ MMW. By measuring the power peaks shown, it is possible to use the presented MMW as a biosensor.

An example of what the output signal at the end of our 4th order Si${}_{3}$N${}_{4}$ MMW sensor would look like is given in Figure 9. By measuring the power at the five positions demonstrated (*x* = $\left\{-\frac{2}{5}W,-\frac{W}{5},0,\frac{W}{5},\frac{2}{5}W\right\}$), marked as *P*${}_{0}$, *P*${}_{1}^{\left(+\right)}$, *P*${}_{1}^{\left(-\right)}$, *P*${}_{2}^{\left(+\right)}$ and *P*${}_{2}^{\left(-\right)}$, it is possible to calculate the *I* signal and use the MMW as a biosensing device.

## 5. Conclusions

In this work, we presented four original contributions: first, a generalization of the concept of BiMW and TriMW sensors, developing new criteria and guidelines for the design of higher-order MMW sensors, as well as exposing its fundamental mechanism of operation in more detail; second, a novel solution for efficiently exciting the desired modes in the multimode section, while maintaining very precise control over the power distribution between them and simultaneously supressing all undesired modes; third, a new mechanism to detect and interpret the output power signal of the sensor, which is robust to oscilations in the power level of the source; finally, we proposed and numerically demonstrated a high-order (4th) MMW sensor based on Si${}_{3}$N${}_{4}$ technology for applications in biosensing with an estimated LOD of $1.9\times 10^{-7}$ RIU for refractive indexes close to 1.33 and $1.3\times 10^{-7}$ RIU for refractive indexes close to 1.46. Compared to other sensors of the same class, the proposed device has shown superior intristic bulk sensitivities, ranging from 23.4% to 58.5% compared to the best designs available in the literature, making it, to the best of our knowledge, the most sensitive multimode interference sensor reported to date.

## Figures and Tables

**Table 1:** Difference in propagation constants (Δβ) for TE${}_{0n}$ modes (Si${}_{3}$N${}_{4}$ channel waveguide with h = 150 nm).

Mode Number (n)	w [nm]	Δβ [$10^{6}$ rad/m]
1	600	1.79
2	900	2.36
3	1300	2.29
4	1600	2.50
5	1900	2.64
6	2200	2.74
7	2500	2.83
8	2900	2.70

**Table 2:** Power distribution for TE${}_{0n}$ modes, for Si${}_{3}$N${}_{4}$ channel waveguide with h = 150 nm.

Mode Number (n)	w [nm]	P${}_{\mathrm{core}}$ TE${}_{00}$ [%]	P${}_{\mathrm{clad}}$ TE${}_{00}$ [%]	P${}_{\mathrm{subs}}$ TE${}_{00}$ [%]
0	600	69.4	13.1	17.5
0	900	70.9	12.2	16.9
0	1300	71.4	11.9	16.7
0	1600	71.5	11.9	16.7
0	1900	71.5	11.8	16.6
0	2200	71.6	11.8	16.6
0	2500	71.6	11.8	16.6
0	2900	71.6	11.8	16.6
**Mode Number (n)**	**w [nm]**	**P${}_{\mathrm{core}}$ TE${}_{0n}$ [%]**	**P${}_{\mathrm{clad}}$ TE${}_{0n}$ [%]**	**P${}_{\mathrm{subs}}$ TE${}_{0n}$ [%]**
1	600	56.1	20.7	23.2
2	900	56.0	20.0	24.0
3	1300	61.7	15.6	22.7
4	1600	62.1	15.8	22.0
5	1900	62.1	15.8	22.1
6	2200	61.6	15.6	22.8
7	2500	60.5	15.2	24.4
8	2900	64.8	15.0	20.2

**Table 3:** Difference in propagation constants (Δβ) for TE${}_{0n}$ modes (Si${}_{3}$N${}_{4}$ channel waveguide with h = 300 nm).

Mode Number (n)	w [nm]	Δβ [$10^{6}$ rad/m]
1	500	2.47
2	750	3.25
3	1000	3.62
4	1200	4.12
5	1500	3.98
6	1800	3.87

**Table 4:** Power distribution for TE${}_{0n}$ modes, for Si${}_{3}$N${}_{4}$ channel waveguide with h = 300 nm.

Mode Number (n)	w [nm]	P${}_{\mathrm{core}}$ TE${}_{00}$ [%]	P${}_{\mathrm{clad}}$ TE${}_{00}$ [%]	P${}_{\mathrm{subs}}$ TE${}_{00}$ [%]
0	500	90.0	5.1	4.9
0	750	91.4	3.9	4.6
0	1000	91.8	3.6	4.6
0	1200	91.9	3.5	4.6
0	1500	92.0	3.5	4.5
0	1800	92.0	3.5	4.5
**Mode Number (n)**	**w [nm]**	**P${}_{\mathrm{core}}$ TE${}_{0n}$ [%]**	**P${}_{\mathrm{clad}}$ TE${}_{0n}$ [%]**	**P${}_{\mathrm{subs}}$ TE${}_{0n}$ [%]**
1	500	75.4	16.4	8.2
2	750	76.4	15.1	8.5
3	1000	77.0	14.3	8.7
4	1200	72.2	16.2	11.6
5	1500	77.0	12.5	10.5
6	1800	81.9	10.7	7.4

**Table 5:** Comparison of bulk sensitivity per sensor length between MMW interference devices in the literature.

Sensor Characteristics	$S_{\mathrm{bulk}}/L$ [rad·RIU${}^{-1}\cdot$μm${}^{-1}$]	Reference, Year
4th order Si${}_{3}$N${}_{4}$ channel WG	1.787	This work
BiMW Si${}_{3}$N${}_{4}$ rib WG	0.849	Zinoviev et al., 2011 [27]
BiMW Si${}_{3}$N${}_{4}$ rib WG with grating couplers	0.963	Duval et al., 2012 [30]
TriMW Si${}_{3}$N${}_{4}$ channel WG	1.131	Ramirez et al., 2015 [28]
Grating-assisted TriMW ma-P 1205 channel WG	0.859	Liang et al., 2018 [29]
Taper-coupled BiMW Si${}_{3}$N${}_{4}$ rib WG	0.901	Grajales et al., 2019 [16]
TriMW SU-8 channel WG with DSMW excitation	0.62	Ebihara et al., 2019 [18]

**Table 6:** Optimized BC-SMW/DC-SMW excitation parameters.

w${}_{\mathrm{SMW}}$ [nm]	w${}_{\mathrm{MMW}}$ [m]	L${}_{\mathrm{DC}}$ [m]	g${}_{1}$ [nm]	L${}_{\mathrm{BC}-\mathrm{SMW}}$ [m]	g${}_{2}$ [nm]
110	1.2	6.5	300	17.4	210

**Table 7:** Power distribution between the modes of the MMW sensor relative to the input power in the DC-SMWs.

Mode	Power [%]
TE${}_{00}$	47.93
TE${}_{01}$	0.00
TE${}_{02}$	0.03
TE${}_{03}$	0.00
TE${}_{04}$	47.91

## Data Availability

The authors confirm that the data supporting the findings of this study are available within the article.
